# VolumE maNagement Under body composition monitoring in critically ill patientS on CRRT: study protocol for a randomized controlled trial (VENUS trial)

**DOI:** 10.1186/s13063-018-3056-y

**Published:** 2018-12-12

**Authors:** Hyung Jung Oh, Jung Nam An, Sohee Oh, Harin Rhee, Jung Pyo Lee, Dong Ki Kim, Dong-Ryeol Ryu, Sejoong Kim

**Affiliations:** 1 Ewha Institute of Convergence Medicine, Ewha Womans University Mokdong Hospital, Seoul, Republic of Korea; 2 Research Institute for Human Health Information, Ewha Womans University Mokdong Hospital, Seoul, Republic of Korea; 3 Department of Critical Care Medicine, Seoul National University Boramae, Medical Center, Seoul, Republic of Korea; 4 Department of Internal Medicine, Seoul National University Boramae, Medical Center, Seoul, Republic of Korea; 5 Department of Biostatistics, Seoul National University Boramae, Medical Center, Seoul, Republic of Korea; 6 Department of Internal Medicine, Pusan National University School of Medicine, Busan, Republic of Korea; 7 Biomedical Research Institute, Pusan National University Hospital, Busan, Republic of Korea; 8 Department of Internal Medicine, Seoul National University College of Medicine, Seoul, Republic of Korea; 9 Department of Internal Medicine, School of Medicine, Ewha Womans University, Seoul, Republic of Korea; 10 Tissue Injury Defense Research Center, Ewha Womans University, Seoul, Republic of Korea; 11 Department of Internal Medicine, Seoul National University Bundang Hospital, 82, Gumi-ro 173 beon-gil, Bundang-gu, Seongnam-si, Gyeonggi-do 13620 Republic of Korea

**Keywords:** Fluid balance, Bioimpedance analysis (InBody S10 (InBody®) Seoul, Korea), Continuous renal replacement therapy (CRRT)

## Abstract

**Background:**

Despite recent technical advances in the management of acute kidney injury (AKI), such as continuous renal replacement therapy (CRRT), intensive care unit mortality is still high, at approximately 40 to 50%. Although several factors have been reported to predict mortality in AKI patients, fluid overload (FO) during CRRT is a well-known predictor of patient survival. However, FO has been mostly quantified as an arithmetical calculation and determined on the basis of the physicians’ perception. Even though such quantification and assessment provides an easy evaluation of a patient’s fluid status and is a simple method, it is not applicable unless a detailed record of fluid monitoring is available. Furthermore, the method cannot differentiate excess water in individual water compartments but can only reflect excess total body water. Bioimpedance analysis (BIA) has been used to measure the nutritional component of body composition and is a promising tool for the measurement of volume status. However, there has been no prospective interventional study for fluid balance among CRRT-treated AKI patients using BIA. Therefore, we will investigate the usefulness of fluid management using the InBody S10 (InBody®, Seoul, Korea), a BIA tool, compared with that of generally used quantification methods.

**Methods/design:**

This will be a multicenter, prospective, randomized controlled trial. A total of 244 patients undergoing CRRT treatment will be enrolled and randomly assigned to receive either to InBody S10-guided management or to fluid management based only on clinical information for 7 days. The primary outcome is to compare the rate of euvolemic status 7 days after the initiation of CRRT, with a secondary outcome being to compare the 28-, 60-, and 90-day mortality rates between the two groups.

**Discussion:**

This will be the first clinical trial to investigate the effect of using BIA-guided fluid management to achieve euvolemia in CRRT-treated AKI patients.

**Trial registration:**

ClinicalTrials.gov, ID: NCT03330626. Registered on 6 November 2017.

**Electronic supplementary material:**

The online version of this article (10.1186/s13063-018-3056-y) contains supplementary material, which is available to authorized users.

## Background

Continuous renal replacement therapy (CRRT) has been established to manage the excretion of toxins and the balance of electrolytes and fluids in critically ill patients with acute kidney injury (AKI) [1–4]. Despite technical advances in the management of AKI over the past few years [5, 6], intensive care unit (ICU) mortality is still high at approximately 40 to 50% [7–10]. Although several factors have been reported to predict mortality in AKI patients [7, 8], fluid overload (FO) at the initial time of CRRT is a well-known predictor of patient survival [7, 11–16]. A few recent studies have examined the effect of fluid accumulation on mortality: Neyra et al. [14] showed that higher cumulative fluid balance during 72 h of ICU admission was independently associated with hospital mortality regardless of AKI or chronic kidney disease (CKD) presence; in the Randomized Evaluation of Normal vs. Augmented Level of Replacement Therapy (RENAL) study, a negative mean daily fluid balance was consistently related to improved clinical outcomes [15]; and Garzotto et al. [16] emphasized the association between the severity and speed of fluid accumulation and ICU mortality.

However, FO has been mostly quantified as an arithmetical calculation: the difference between the sum of daily fluid intake and total output adjusted by body weight and determined based on the physician’s perception. Even though such quantification and assessment is an easy mechanism to evaluate a patient’s fluid status and is a basic method, it is not applicable unless a detailed record of the fluid monitoring is available, and it cannot differentiate water excess in individual water compartments; instead, the method can only reflect excess total body water (TBW) [17].

Bioimpedance analysis (BIA) has been used to measure the nutritional part of body composition, such as fat mass or fat-free mass (FFM), in diverse conditions [18, 19]. However, it has been used as a promising tool for the measurement of volume status [20]. With the electrical properties of body tissues, multifrequency-BIA (MF-BIA) differentiates extracellular water (ECW) or intracellular water (ICW) from TBW using different frequencies: 0, 1, 5, 50, 100, 200, 500 or 1000 kHz [21, 22]. To date, several studies have demonstrated the accuracy and clinical usefulness of MF-BIA in chronic hemodialysis or peritoneal dialysis patients [23–27]. Moreover, in critically ill patients, MF-BIA has been useful in assessing volume status [28] and net fluid removal using CRRT to successfully reduce TBW, ECW, and ICW [29]. Rhee et al. [17] investigated the effect of MF-BIA-defined volume status on the mortality of critically ill patients with AKI; the authors demonstrated that MF-BIA-defined excess volume parameters adjusted for the square of the patient’s height (H^2^), such as TBW/H^2^ and ICW/H^2^, were independently associated with higher in-hospital mortality in male patients with AKI undergoing CRRT. However, there has been no prospective interventional study for fluid balance among CRRT-treated AKI patients using the abovementioned BIA. Therefore, in this study, we will investigate the usefulness of fluid management using InBody S10 (InBody®, Seoul, Korea), a BIA tool, and compared with a generally used quantification method.

## Methods/design

### Hypothesis

BIA-guided fluid management will reach an euvolemic status in patients who are treated with CRRT better than fluid management guided by a generally used quantification method, which is calculated by the difference between the sum of daily fluid intake and total output adjusted by body weight and determined based on the physician’s perception. Moreover, the patients who achieve a euvolemic status 7 days after the initiation of CRRT will have better clinical outcomes based upon 28-, 60-, and 90-day mortality than those of patients who are still in a higher-volume status at the same time.

### Study design

**Fig. 1:**
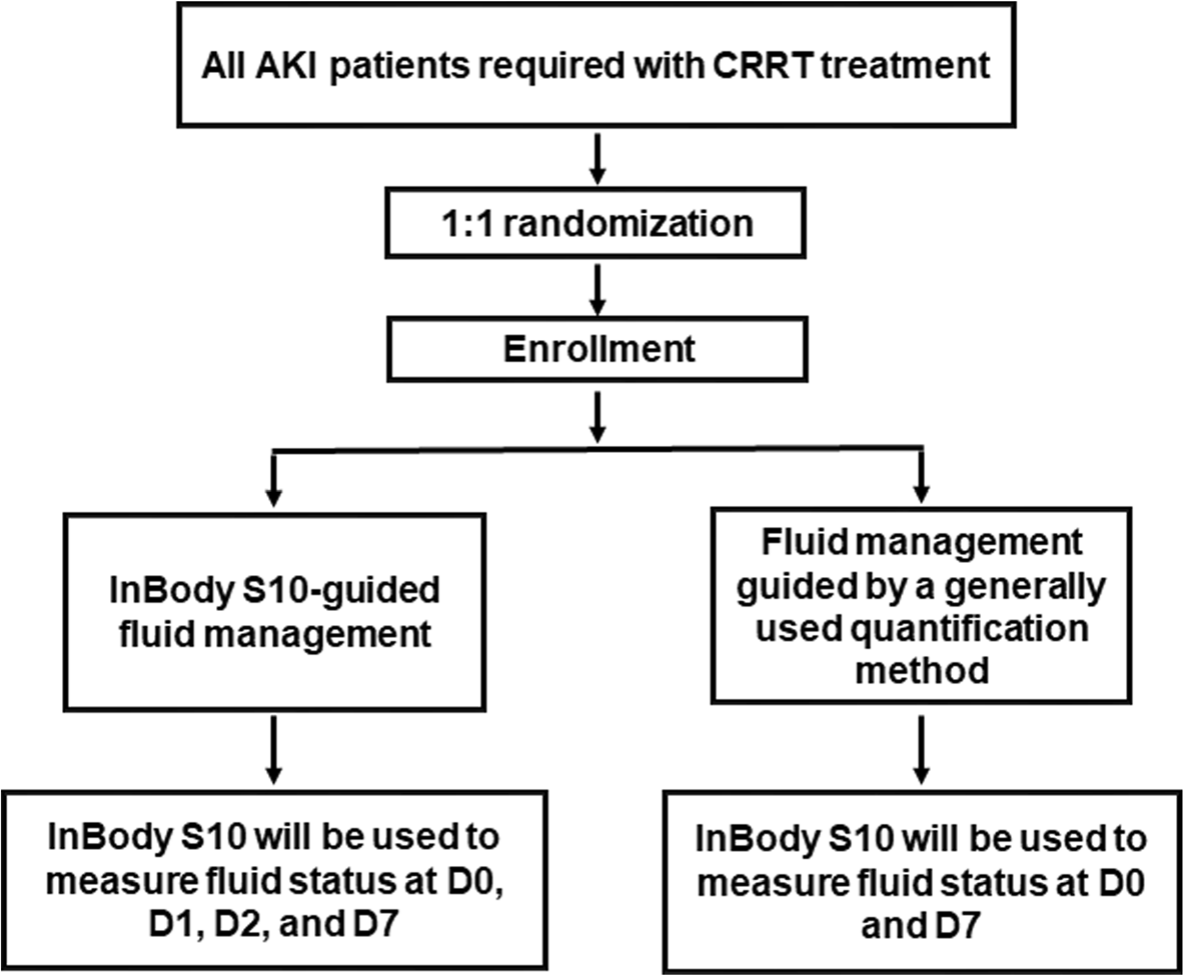
The overall study algorithm. After enrollment, InBody S10 (InBody®, Seoul, Korea) will be used to measure fluid status at 0, 1, 2, and 7 days after the initiation of the continuous renal replacement therapy (CRRT) in InBody S10-based fluid management group, but at 0 and 7 days in fluid management guided by a generally used quantification method. Abbreviations: *AKI* acute kidney injury, *CRRT* continuous renal replacement therapy, *D0* the day of CRRT initiation, *D1*, *2 and 7* 1, 2 and 7 days from the day of CRRT initiation, respectively

**Fig. 2:**
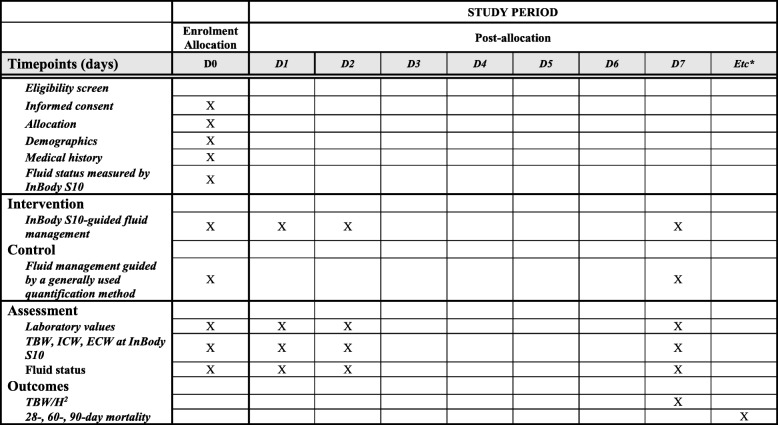
Schedule of enrollment, interventions, and assessments according to the Standard Protocol Items: Recommendations for Interventional Trials (SPIRIT) guideline. Abbreviations: *TBW* total body water, *ICW* intracellular water, *ECW* extracellular water, *TBW/H*^*2*^ total body water/height^2^

This will be a multicenter, prospective, parallel-group, open-label, randomized controlled trial. It is an investigator-initiated clinical trial. We have followed the Standard Protocol Items: Recommendations for Interventional Trials (SPIRIT) 2013 Statement which defines standard protocol items for clinical trials (Additional file 1). The overall study algorithm is depicted in Fig. 1 and the SPIRIT and study schedule are given in Fig. 2. After enrollment, InBody S10 (InBody®, Seoul, Korea) will be used to measure fluid status at 0, 1, 2, and 7 days after the initiation of the CRRT in InBody S10-based fluid management group, but at 0 and 7 days in fluid management guided by a generally used quantification method. Physicians will not know the results of the InBody S10 measurement. Instead, another investigator will measure the InBody S10 and record the results. In the treatment group, the fluid monitoring will be managed according to Table 1, while the fluid balancing of the control group will be conducted based on the clinical information obtained by the physician, such as body weight, hemodynamic stability and daily intake and output. Clinical information including hemodynamic monitoring and laboratory data will be collected on the same days, and we will investigate the 28-, 60-, and 90-day survival status.

**Table 1:** The guideline of fluid management for treatment group

TBW/H^2^ at D0, D1, D2	Target fluid removal amount (L/H^2^/day)	
13–14	−0.5	Or 1 L/day
14–15	−0.5	
15–16	−0.6	
16–17	−0.6	
17–18	−0.7	
18–19	−0.7	
19–	−0.7	

Fluid removal will be determined based on the TBW/H^2^ for 3 days (D0, D1, D2). However, fluid will be removed by 1 L/day if calculated fluid removal from the TBW/H^2^ is over 1 L/day

### Study participants and measurements

All patients will be selected from among patients who must be treated with CRRT in four tertiary hospitals in Korea (Seoul National University Bundang Hospital, Seoul National University Hospital, Seoul National University Boramae Medical Center, and Ewha Womans University Mokdong Hospital). Patients aged over 18 years who require CRRT for AKI will be screened for study participation according to the inclusion and exclusion criteria presented in Table 2.

**Table 2:** Inclusion and exclusion criteria

Inclusion criteria	
Patients will be eligible for *inclusion* in the study if *all* the following criteria are met:	
1. The treating clinician believes that the patient requires continuous renal replacement therapy for acute kidney injury	
2. The treating clinicians anticipate treating the patient with continuous renal replacement therapy for at least 72 h	
3. Informed consent has been obtained	
4. The patient fulfills one of the following clinical criteria for initiating continuous renal replacement therapy:	
Urine output < 100 mL/6 h that has been unresponsive to fluid resuscitation measures	
K^+^ > 6.5 mmol/L	
pH < 7.2	
Urea > 25 mmol/L	
Clinically significant organ edema in the setting of acute kidney injury	
5. Patients who are over 5% of fluid overload or their total body water/height^2^ ≥ 13 L/m^2^	
Exclusion criteria	
Patients will be *excluded* from the study if, in the opinion or knowledge of the responsible clinician *any* of the following criteria are present:	
1. Patient age is < 18 years	
2. Death is imminent (< 24 h)	
3. There is a strong likelihood that the study treatment will not be continued in accordance with the study protocol.	
4. The patient has been treated with continuous renal replacement therapy or other dialysis previously during the same hospital admission.	
5. The patient has been on maintenance dialysis prior to the current hospitalization.	
6. Any other major illness that, in the investigator’s judgment, will substantially increase the risk associated with the subject’s participation in this study.	

### Randomization

A research coordinator will perform the randomization. A list of random numbers will be generated by an independent statistician. Eligible participants will be randomly assigned 1:1 to either the control group (fluid management based on the clinical information alone) or the treatment group (InBody S10 (InBody®, Seoul, Korea)-guided fluid management alone). Randomization will be stratified based on the institution and will utilize a randomized block design.

### Outcome measures

The primary outcome is to compare the rate of euvolemic status at 7 days from the CRRT start. The euvolemic status will be considered different when TBW/H^2^ between the day of CRRT initiation (D0) and 7 days from the day of CRRT initiation (D7) is less than − 2.1 or TBW/H^2^ at D7 is less than 13 L/m^2^. According to Rhee’s recent study, the group reaching TBW/H^2^ < 13 L/m^2^ had a significantly enhanced survival rate compared with that of the group that could not reach TBW/H^2^ < 13 L/m^2^ [17]. Moreover, our further study has revealed that the survival rate was significantly increased when the difference in TBW/H^2^ between CRRT initiation and at 7 days from CRRT start (TBW/H^2^_at 7days_ – TBW/H^2^_at CRRT initiation_) was less than − 2.1 L/m^2^, even though it was not sufficient to reach TBW/H^2^ < 13 L/m^2^ (data not shown). Therefore, we will also consider the euvolemia as TBW/H^2^_at 7days_ – TBW/H^2^_at CRRT initiation_ < − 2.1 L/m^2^ in this study. The secondary outcome is to investigate the 28-, 60-, and 90-day mortality rates in the control and treatment groups and to compare the mortality rates between the groups that reach euvolemic status or not for sub-analysis.

### Assessment of the fluid status

For participants in the control group, clinical information obtained by the physician is the standard of judgment. However, the participants will be measured by InBody S10 (InBody®) at D0 and D7, and the results will be recorded. Clinical information will be composed of four items: body weight in the morning; hemodynamic stability; absence of symptoms and signs of hypervolemia (dyspnea, edema, and crackle) or hypovolemia (dizziness and orthostatic hypotension); and daily intake and output. For participants in the treatment group, InBody S10 (InBody®) will also be measured at 9:00 a.m. on D0, D1, D2, and D7. Table 1 shows the guideline of fluid management for the treatment group. However, both the physicians and participants will be blinded to the results of InBody S10 (InBody®) analysis.

InBody S10 (InBody®) is a medical device that has been approved by the US Food and Drug Administration as an impedance body-fat analyzer. The device provides the following parameters: TBW, ICW, ECW, segmental water values, ECW ratio (ECW/TBW), FFM, soft lean mass (SLM), segmental lean mass, body fat mass (BFM), percentage body fat (PBF), and TBW/FFM.

The BIA method calculates body composition using the resistance value (impedance) that appears due to the difference in electrical conductivity according to the biological characteristics of each tissue. Electrical conductivity is proportional to the amount of water and electrolytes, and the water content of fat tissue is relatively smaller than that of other tissues, which leads to a decrease in electrical conductivity when the fat content increases. Among the components of the body, body water is the component that passes an electrical current, so the volume of water in the body can be obtained by measuring the resistance value obtained from body water [21].

### Clinical and laboratory evaluations

Physical examination, comorbidity and medication will be reviewed. Laboratory evaluations, including complete blood cell counts (CBC), electrolytes, creatinine, protein, albumin, calcium, phosphorous, total cholesterol, triglyceride, low-density lipoprotein cholesterol, high-sensitivity C-reactive protein (hs-CRP), and *N*-terminal prohormone of brain natriuretic peptide (NT-proBNP) will also be performed in the morning in the intensive care unit.

### Safety issues

The InBody S10 (InBody®) method approved by the US Food and Drug Administration has been used in clinical practice. Applying weak alternating currents into the body is known to not be harmful.

### Sample size calculations

No previous report has evaluated the effect of InBody S10 (InBody®)-guided fluid management on CRRT-treated patients. We will randomly divide the enrolled participants equally into two groups. Assuming a 19% difference in the rate of achieving euvolemic status between the two groups with 0.8 of power, two-sided, and 0.05 of alpha, 97 participants will be allocated in each group. However, 122 participants will be required to enroll in each group due to a 20% withdrawal rate.

### Statistical analyses

The statistical analyses will be conducted both on a per-protocol (PP) and an intention-to treat (ITT) basis. For PP analysis, all participants who complete the study will be included to evaluate the primary and secondary outcomes. For the ITT analysis, all participants who are enrolled and randomized to one of the two groups and who complete the first visit will be included. Basic statistics will be reported in terms of the mean ± SD for continuous variables, or as percentages for categorical variables. Differences between groups will be analyzed using Student’s *t* test for continuous variables and the *χ*^2^ test or Fisher’s exact test for categorical variables. The primary outcome will be compared with Student’s *t* test. An analysis of covariance (ANCOVA) will be used to analyze the primary outcome as the secondary analysis to adjust the baseline value. Multivariate Cox proportional hazard regression models will be used to analyze the time to mortality. Although the institutions are mainly located in large cities, thereby limiting the likelihood of any important cluster effect, we will be sure to accommodate possible clustering in our models and analysis as required. A value of *P* < 0.05 will be considered statistically significant. All analyses will be performed using SPSS Statistics software (v21.0; IBM Corporation, Armonk, NY, USA).

### Ethics approval

The study will be performed in accordance with the Declaration of Helsinki, as amended by the 59th World Medical Association General Assembly in 2008. All the participants will provide signed, informed, written consent, stating that participation is voluntary and can be withdrawn at any time. Approval for the study has been obtained from the Institutional Review Board of Seoul National University Bundang Hospital (B-1702/383–003), Seoul National University Hospital (J-1705-080-855), Seoul National University Boramae Medical Center (20,170,516/20–2017-2/062), Ewha Womans University Mokdong Hospital (EUMC 2017–05–049-008) (Ethical Approval Document). The trial protocol has been registered at https://clinicaltrials.gov/ct2/show/NCT03330626 (NCT03330626). Moreover, this research was supported by a grant from the Korea Health Technology R&D Project through the Korea Health Industry Development Institute (KHIDI), funded by the Ministry of Health and Welfare, Republic of Korea (grant number: HI17C1827) (Funding Documentation).

## Discussion

CRRT is a generally used tool to manage fluid balance among the critically ill patients with AKI when they have medically refractory overhydration [1–4]. Moreover, some recent studies have emphasized the effects of cumulative fluid balance on mortality among CRRT-treated AKI patients [14–16]. However, FO has been mostly quantified as an arithmetical calculation using the following equation: (The sum of daily fluid intake − total output) / (Body weight). This condition has been determined based on the physician’s perception. However, it is not applicable unless a detailed record of fluid monitoring is available, and it cannot differentiate water excess in individual water compartments [17], although such quantification and assessment represents an easy and basic method to evaluate a patient’s fluid status.

InBody S10 (InBody®) is a medical device using MF-BIA and has been approved by the US Food and Drug Administration as an impedance body-fat analyzer that also provides analysis of individual water compartments, such as TBW, ICW, ECW, segmental water values, and ECW ratio (ECW/TBW).

MF-BIA has proven to be useful for assessing volume status in critically ill patients [28, 29] and Rhee et al. [17] showed the effect of MF-BIA-defined volume status on the mortality of critically ill patients with AKI using InBody S20 (InBody®). As there has not yet been a prospective interventional study of fluid balance among CRRT-treated AKI patients using the abovementioned BIA, we will investigate the usefulness of fluid management using the BIA device InBody S10 (InBody®) and compare the results with a generally used quantification method.

To our knowledge, this will be the first multicenter, prospective, randomized controlled trial to assess whether InBody S10-guided volume management is better able to reach a euvolemic status at 7 days after the initiation of CRRT than the quantitation method for volume management is. The endpoint of the VENUS (VolumE maNagement Under body composition monitoring in critically ill patientS on CRRT) study may be of the utmost importance to healthcare providers.

So far, there is no definite index for determining euvolemic status. Instead, most physicians determine their patients’ volume status based on clinical information, such as intake and output, chest radiographic examination, and physical examination. Therefore, the fluid balance of patients may be managed differently based on the physicians’ opinions, even with the same patients. The abovementioned study by Rhee et al. [17] suggested that TBW/H^2^ and ICW/H^2^ were independently associated with higher in-hospital mortality in male patients with AKI undergoing CRRT, and the group reaching TBW/H^2^ < 13 L/m^2^, which was 13% of total enrolled patients, had a significantly increased level of survival compared to that of other patients. Thus, we will define the euvolemia as the status of TBW/H^2^ < 13 L/m^2^. In addition, our study of fluid removal has revealed that the difference in TBW/H^2^ between CRRT initiation and at day 7 of CRRT,[(BW/H^2^_at 7days_ – TBW/H^2^_at CRRT initiation_), especially when it was greater than − 2.1 L/m^2^, was significantly associated with an increased mortality rate, even though it did not reach TBW/H^2^ < 13 L/m^2^ (data not shown). Therefore, we will also consider another measure of euvolemia in this study, namely TBW/H^2^_at 7days_ – TBW/H^2^_at CRRT initiation_ < − 2.1 L/m^2^.

There is a chance that patients within the same institution will be correlated more closely than patients at different institutions will. To avoid this issue, we will investigate for the possible clustering of patients at the recruitment institution level and will accommodate such clustering in our models and analysis as required. We have also conservatively calculated the sample size, assuming a high drop-out rate, to overcome this potential problem.

There are a few limitations in this study. First, this study will be investigated in Korean Tertiary Hospitals, which means that the results will require cautious interpretation concerning ethnicity. Second, we have defined euvolemia arbitrarily, although this definition was based on recent data and upon our own study. However, this study is the first study, to the best our knowledge, to determine guidelines for the fluid balance in CRRT-treated critically ill AKI patients using a prospective randomized controlled trial. We expect that the endpoint of the VENUS study will provide the utmost information to healthcare providers.

In summary, the VENUS study is the first prospective, randomized controlled trial to evaluate the clinical usefulness of InBody S10-guided fluid management in CRRT-treated critically ill AKI patients. The aim of this study is to identify more objective guidelines to manage volume status in CRRT-treated, critically ill AKI patients.

## Trial status

This trial is ongoing. Participants are currently being recruited.

## Additional file

Additional file 1: Standard Protocol Items: Recommendations for Interventional Trials (SPIRIT) 2013 Checklist: recommended items to address in a clinical trial protocol and related documents. (DOC 124 kb)
